# Epigenetic modulator UVI5008 inhibits MRSA by interfering with bacterial gyrase

**DOI:** 10.1038/s41598-018-31135-9

**Published:** 2018-09-03

**Authors:** Gianluigi Franci, Veronica Folliero, Marcella Cammarota, Carla Zannella, Federica Sarno, Chiara Schiraldi, Angel R. de Lera, Lucia Altucci, Massimiliano Galdiero

**Affiliations:** 1 Dipartimento di Medicina Sperimentale, Università degli Studi della Campania “Luigi Vanvitelli”, Napoli, Italy; 2 Dipartimento di Medicina di Precisione, Università degli Studi della Campania “Luigi Vanvitelli”, Napoli, Italy; 3 Departamento de Química Orgánica, Facultade de Química, Universidade de Vigo, CINBIO and IBIV, Vigo, Spain

## Abstract

The impact of multi-drug resistant bacterial strains on human health is reaching worrisome levels. Over 2 million people are infected by resistant bacteria, and more than 700,000 people die each year because of the continuous spread of resistant strains. The development of new antibiotics and the prudent use of existing ones to prolong their lifespan require a constant effort by drug industries and healthcare workers. The re-purposing of existing drugs for use as antimicrobial agents would streamline the development of new antibacterial strategies. As part of this effort, we screened a panel of drugs previously characterized to be epigenetic modulators/pro-apoptotic/differentiative drugs. We selected a few compounds that alter Gram-positive growth. Among these, UVI5008, a derivative of the natural compound psammaplin A (Psa_A), was identified. The interaction of Psa_A with the DNA gyrase enzyme has been shown, and here, we hypothesized and confirmed the gyrase-specific activity by biochemical assays. UVI5008 exhibited growth inhibition activity against *Staphylococcus aureus* via structural modification of the cell wall, which was observed by SEM electron microscopy. Based on our findings, we propose UVI5008 as an alternative antibacterial compound against methicillin-resistant (Met.R) *S*. *aureus* strains.

## Introduction

The application of antibiotics for human health, which began in the 1940s, has brought about huge scientific and social transformations in the medical field^1^. As a result of the introduction of antibiotics in patient care, several infections, even some deadly bacterial diseases, can be effectively cured. In the following years, several advances led to the identification of additional semi-synthetic and synthetic antibacterial agents that successfully reached clinical application^2^. Antibiotics represent a priority in the worldwide healthcare system^3^. Today, we are facing the global rise of highly resistant bacteria, and therefore, new antimicrobial identification as well new combinatorial strategies is of crucial importance^4,5^. Many bacteria are resistant against several types of antibiotics and are commonly defined as superbugs^6^. According to the Centers for Disease Control (CDC, USA), each year, these superbugs cause more than 700,000 deaths globally, and this figure is predicted to reach 10 million in 2050. The latest WHO data showed more than 400,000 new cases of multidrug-resistant tuberculosis in 2015^3^. The CDC has previously highlighted the increasing number of patients who are suffering from common infections generated by multi-resistant bacteria^7,8^. Hospitals are an astonishing hot-spot of highly resistant bugs^9,10^. Bacteria with high resistance to antibiotics are responsible for the most severe hospital outcomes and, either directly and indirectly, for increasing healthcare costs. A plethora of natural events can cause or contribute to the bacterial acquisition of resistance to antibiotics. Either extensive and/or incongruous use of antibiotics may result in a strong selective pressure leading to the emergence of drug-resistant bacteria^11,12^. Several studies indicate that bacteria residing in biofilms can easily acquire genetically transmissible elements due to the physical proximity of individual cells. This renders biofilms an ideal place for the onset of antibiotic resistant pathogens. In addition, biofilm-associated bacteria are often shielded from the action of antibiotics. The antibiotics do not efficiently permeate the biofilm and even when they do, they are not effective against dormant cell subpopulations^13^.

The most dangerous microorganisms in terms of human diseases have been grouped under the term “ESKAPE” pathogens (*Enterococcus faecium*, *Staphylococcus aureus*, *Klebsiella pneumoniae*, *Acinetobacter baumannii*, *Pseudomonas aeruginosa*, and *Enterobacter species*). These bacteria are known to “escape” the inhibition of several antimicrobial drugs^14^. The pathogens leading to increased mortality include carbapenem-resistant Enterobacteriaceae (CRE), P. *aeruginosa*, *A*. *baumannii*, methicillin-resistant *S*. *aureus* (MRSA) and vancomycin-resistant *Enterococcus*^3^. All of the current state-of-art developments in organ transplantation, chemotherapy, and surgery are seriously threatened by the incumbent introduction of these aggressive superbugs.

In this scenario, optimized versions of existing antibiotics or novel synthetic compounds have been pursued, but natural products still represent a major source of antibiotics that work effectively, since they have been moulded by evolution to work on natural targets. Several epigenetic modulators are natural compounds; therefore, we investigated the antibiotic potential of some of these modulators.

Epigenetic modulators represent a widespread class of small molecules that regulate chromatin by modifying histone and non-histone targets tuning gene expression^15^. Recent evidence has reported the activity of some of these compounds not only on eukaryotic cells but also on microbial pathogens^16,17^. Histone deacetylase (HDAC) inhibitors have been reported to increase the replication rate of several viruses, thus triggering the mechanism(s) of latency exit^18^. On this basis, clinical trials have been initiated for the use of SAHA (pan-HDAC inhibitor) treatment for patients with human immunodeficiency virus (HIV) in combination with antiretroviral therapy (cART), as reported on the official US government clinical trials website. Given that bacteria do not have histones^19^, many of the targets and enzymes that can be altered in eukaryotic cells to induce epigenetic changes do not exist in bacteria^20^. Nevertheless, different DNA methyltransferases (DNMTs) target unique DNA sequences, where they attach to methyl groups on cytosine and adenine bases near the promoter regions, causing variations in gene expression. It is widely accepted in the field that DNMT enzyme inhibitors alter normal bacterial gene expression^21^. We hypothesized that bacterial methyltransferase may be targeted by these epigenetic modulators, which may therefore suggest a novel class of antibiotics. To test our hypothesis, we screened 12 modulators, some of which have single or multiple epigenetic targets. From these studies, we identified an indole derivative of psammaplin A (Psa_A) named UVI5008, known in the literature as a novel epigenetic modulator for cancer treatment^22^, which showed significant inhibitory activity against MRSA.

## Results

### Antibacterial screening

**Figure 1:**
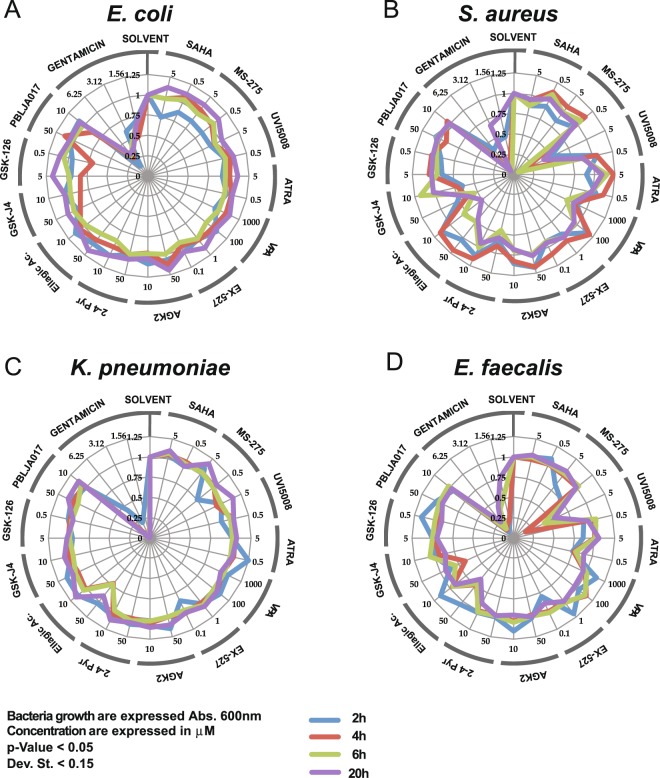
Radar graph representation of the effect of epigenetic compounds on two Gram-negative (*E*. *coli*, *K*. *pneumoniae*) and two Gram-positive (*S*. *aureus*, *E*. *faecalis*) bacterias. Compounds and their concentrations (expressed in μM) are reported on the external radar graph circle. All absorbance values were recorded and converted to % of inhibition compared to controls. The results are representative of three independent experiments, and stringent statistical filters were applied

Twelve different compounds at 2 different concentrations were screened as potential inhibitors of bacterial growth (Table 1 in supplementary files)^23^. Results are reported in a radar graph (Fig. 1) as the percentage of growth of the treated samples compared to the controls. The greater the bacterial growth inhibition was, the closer the sample is to the centre of the graph (Fig. 1A–D). As seen in Fig. 1, gentamicin (positive control) inhibited bacterial growth as described in the figure legend. The selected compounds were not effective against Gram-negative strains (Fig. 1A–C). A few of the compounds were effective at inhibiting Gram-positive growth in a dose-dependent manner (Fig. 1B–D). Of the 12 molecules tested, 5 drugs inhibited Gram-positive bacteria. Valproic acid (VPA), 2-4-pyridinedicarboxylic acid (2–4 Pyr), GSK-126, ellagic acid, and UVI5008 demonstrated antimicrobial activity. UVI5008 is an epigenetic modulator, while the remaining compounds have anticancer and/or cell-differentiating properties^23–26^. UVI5008 was selected for its high potency as an antimicrobial and its potentially novel mechanism of action.

### UVI5008 growth inhibition of MSSA and MRSA

**Figure 2:**
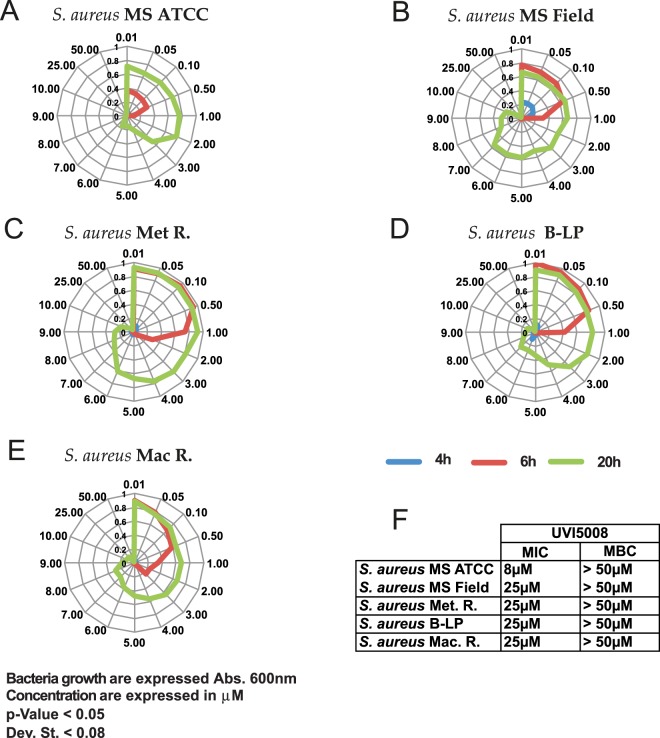
Radar graph representation of bacteria growth at 600 nm absorbance for ATCC MSSA **(A)**, field MSSA **(B)**, Met.R *S*. *aureus* **(C)**, Beta-lactamase producer *S*. *aureus* **(D)**, and macrolide resistant *S*. *aureus* **(E)** at 4 h, 6 h, and 20 h of incubation. Table of MIC and MBC values for the respective *S*. *aureus* strains **(F)**.

To evaluate the minimum inhibitory concentration (MIC) of UVI5008 that results in 100% inhibition of methicillin-sensitive *S*. *aureus* (MSSA), we obtained a dose-response curve from 0.01 μM to 50 μM (Fig. 2).

ATCC MSSA was compared to field isolated (FI) MSSA (Fig. 2A,B). The results are shown in linear graphs. Parameters plotted in the graph were based on the relative absorbance at 600 nm over time. Colour-coding was used to distinguish the time of treatment, with green, violet and blue representing 4, 6 and 20 hours, respectively. ATCC MSSA and FI MSSA were affected differently by the UVI5008 treatment. UVI5008 showed an MIC of 8 μM against the ATCC strain, but the FI MSSA was more resistant to UVI5008 treatment and required a 25 μM dose to reach the MIC.

The UVI5008 inhibition on MSSA was evident, and its effect on *S*. *aureus* resistant strains was also assessed. As reported in Fig. 2, UVI5008 inhibition activity on Met.R, β-lactams producer (B.LP) and macrolid resistant (Mac.R) *S*. *aureus* strains was assessed. The results are shown in panels 2 C, 2D and 2E. UVI5008 treatment at 8 μM was not sufficient to halt growth in overnight (O.N.) incubation. UVI5008 inhibited the MRSA bacterial growth in a dose- and time-dependent manner, and MIC values of 25 μM were obtained for all MRSA strains. These findings are summarized in Fig. 2F. To define the minimum bactericidal concentration (MBC), MSSA and MRSA isolates were plated on brain-heart agar plates post-over night (ON). culture. UVI5008 treatment was maintained at respective concentrations as reported in Supplementary Fig. S1. A more drastic colony formation reduction of MSSA bacteria was observed in a dose-dependent manner compared to MRSA strains. Increasing UVI5008 concentrations led to a consistent decrease in colony formation for both MSSA and MRSA, suggesting that the increase in drug sensitivity of MSSA vs. MRSA also translates to MBC evaluation (Fig. 2F). The MBC was higher than 50 μM in both cases.

### UVI5008 acts against biofilm MRSA producers

Biofilm formation is an important virulence mechanism that helps bacteria resist antibiotics^27^. We therefore asked whether UVI5008 was also active on preformed biofilms. To investigate the role of biofilms in the differences in UVI5008 activity on the ATCC strain and Field Isolated *S*. *aureus* (FI *S*. *aureus*), we first established whether the FI *S*. *aureus* strains formed biofilms using the triphenyl tetrazolium chloride (TTC) reduction assay (Supplementary Fig. 2A). We then determined whether UVI5008 treatment degrades preformed biofilms (Supplementary Fig. 2B). The data show that all FI *S*. *aureus* strains were biofilm producers. In contrast, the ATCC strain did not form a biofilm. We propose that the inability of the ATCC strain to form a biofilm may explain the lower UVI5008 dose required for inhibition compared to the FI strains.

**Figure 3:**
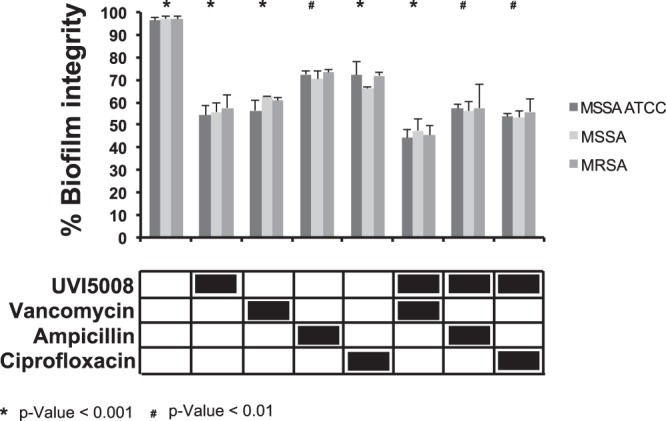
Combinatorial experiments on biofilm degradation mediated by UVI5008 and vancomycin, ampicillin and ciprofloxacin for ATCC SA compared with FI MSSA and MRSA. The results are representative of three independent experiments, and stringent statistical filters were applied (p-value < 0,5).

UVI5008 efficacy on mature biofilms was assessed after a 24 hr incubation with a range of UVI5008 concentrations (from 1 to 50 μM) (Supplementary Fig. 2B). At a dosage of 25 μM, more than 20% biofilm degradation was observed. To determine the biofilm disruption in combination with other commonly used antibiotics, UVI5008 activity was tested in the presence of vancomycin, ampicillin and ciprofloxacin^28–30^. The data showed that the use of UVI5008 in combination with the antibiotics in our assays did not significantly increase the antimicrobial activity. The treatment with vancomycin and UVI5008 resulted in more than 50% biofilm degradation with a slight increase in effect compared to their independent use (Fig. 3). These findings do not show a synergistic effect but do indicate that these two compounds still work when administered together. We propose that a combination of drugs with different mechanisms could be useful to decrease the likelihood of eliciting resistance.

### SAR of UVI5008 and its inhibition on MSSA and MRSA

**Figure 4:**
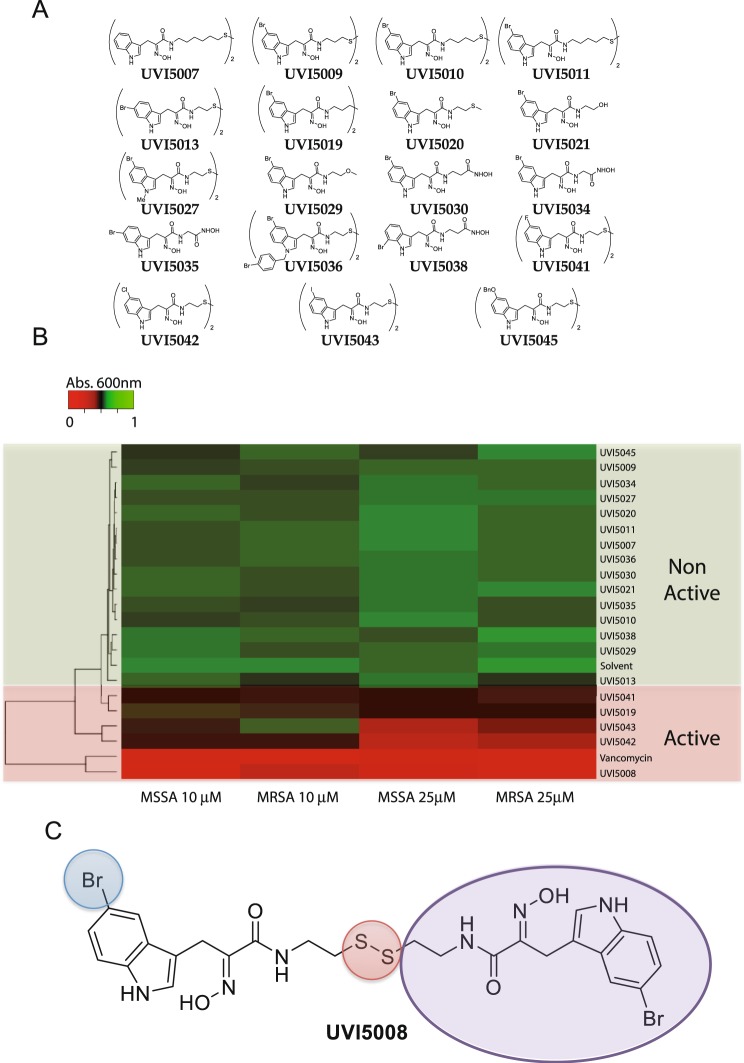
UVI5008 analogues based on SAR **(A)**. Heatmap representation of absorbance acquired for MSSA and MRSA in response to treatment with UVI5008 analogues. The distance between two clusters is the mean distance between all objects of the clusters **(B)**. Structural analysis of UVI5008 characteristics needed for preservation of antibacterial properties **(C)**.

Our experiments demonstrate that UVI5008 has potential as a new drug against Gram-positive bacteria and specifically against *S*. *aureus*. To begin assessing the structure-activity relationship (SAR) of UVI5008, we generated several analogues (Fig. 4A). The UVI5008 structure was modified as previously reported^22^ via functionalization of the C3 indole position with *in situ* generated nitrosoacrylate, followed by protection of the β-indole-α-oximinoesters, saponification, condensation with symmetrical diamines, and deprotection. The different compounds obtained can be grouped as follows (some of the compounds belong to more than one group according to the modifications): (i) compounds with an elongated amidodisulphide hydrocarbon chain (UVI5007, UVI5009, UVI5010 and UVI5011); (ii) monomeric compounds with methyl β-amidosulphide (UVI5020), β-hydroxyamide (UVI5021) and β-alkoxyamide (UVI5029) functionalities and hydroxamic acids (UVI5030, UVI5034, UVI5035 and UVI5038); (iii) compounds bearing halogens and positional variations (UVI5013, UVI5035, UVI5041, UVI5042, and UVI5043); (iv) compounds lacking the disulphide between dimer units (UVI5019, UVI5020, UVI5021, UVI5029, UVI5030, UVI5034, UVI5035 and UVI5038); (v) compounds with modifications at the indole nitrogen (UVI5027, UVI5036 and UVI5045); and (vi) a compound with a benzyloxy group instead of a halogen (UVI5045).

The antimicrobial activities of the analogues were tested against MSSA and MRSA clinical samples at 10 μM and 25 μM (Fig. 4B). Inhibition assays were performed, and the results are presented graphically via heatmap visualization with the tree representing the SAR family. Strong inhibitory activity was achieved at 25 μM for compounds UVI5042 and UVI5043 (Cl and I analogues of UVI5008), which clustered close to UVI5008 and vancomycin. Meanwhile, compounds UVI5041 (fluoro analogue of UVI5008), UVI5013 (positional isomer of UVI5008) and UVI5019 (lacking the disulphide bridge) showed minimal inhibitory activity. Taken together, these results help to identify key structural motifs for preserving UVI5008 activity. The dimeric structure of the compounds with the disulphide bridge in the centre and the Br atom at C5 are both required to preserve the antimicrobial activity of UVI5008 (Fig. 4C).

### UVI5008 molecular mechanism of action against bacteria

**Figure 5:**
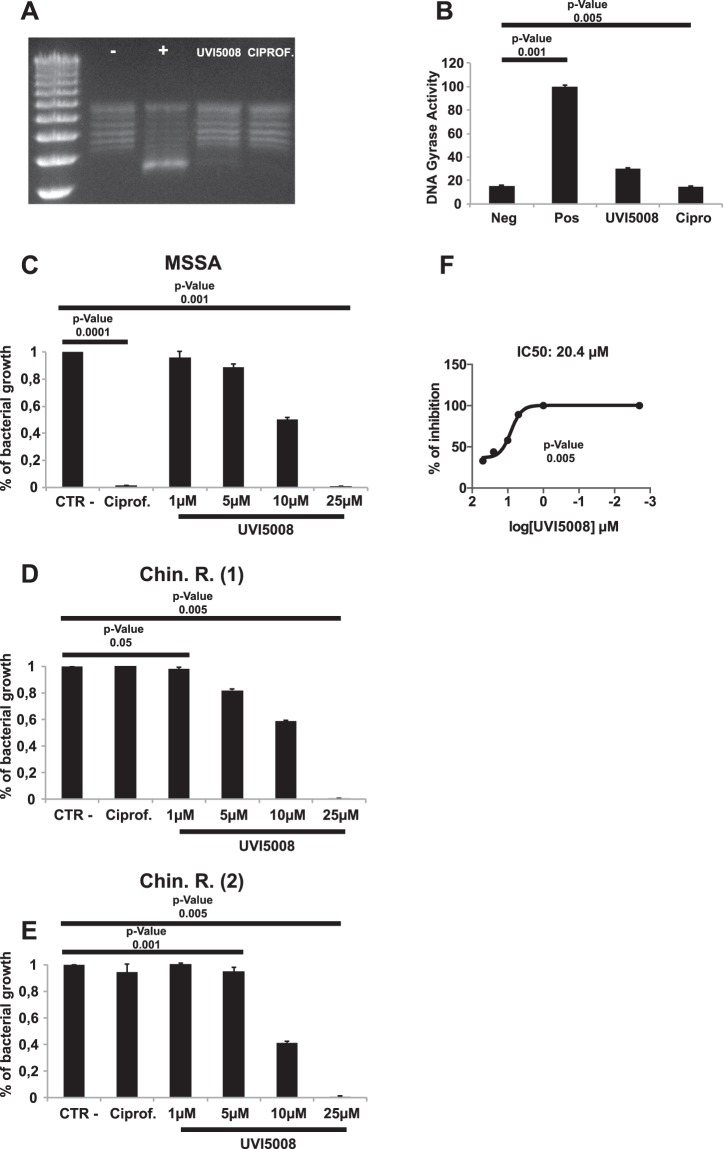
UVI5008 biochemical mechanism of action on *S*. *aureus* recombinant DNA gyrase and supercoiled bacterial DNA **(A)** with the relative quantization obtained via ImageJ software **(B)**. Inhibition activity of UVI5008 on two quinolone-resistant *S*. *aureus* strains compared with the FI MSSA strain **(C**–**E)**. IC_50_ on DNA gyrase was calculated by GraphPad Prism software **(F)**.

DNA gyrase possesses two A subunits and two B subunits. The A subunits induce DNA supercoiling, while the B subunits provide the energy required for the coiling activity by ATP hydrolysis. UVI5008 is a derivative of the natural compound Psa_A^22^, which has been reported to act on *S*. *aureus* DNA gyrase by forming a stable interaction between the compound and DNA gyrase subunit B^17^. To investigate whether UVI5008 acts similarly to Psa_A, we performed a gyrase-specific inhibition assay (Fig. 5A,B). In the gyrase inhibition assay, the substrate is the relaxed circular pBR322 plasmid. Gyrase activity on the relaxed plasmid will result in a supercoiled structure. Supercoiled and relaxed forms can be detected by agarose gel electrophoresis. Inhibition of gyrase activity would result in an increase in relaxed plasmid conformations. Ciprofloxacin was used as the positive control since it blocks gyrase acting on the A subunit^31^. In the presence of UVI5008, the plasmid supercoiled structure formation was reduced by more than 80% (Fig. 5B) with an IC_50_ of 20.4 μM (Fig. 5F).

Figure 5 provides evidence that UVI5008 effectively inhibits the gyrase activity. To further dissect whether UVI5008 acts on DNA gyrase subunit A or subunit B, we performed an inhibition assay on the growth of a quinolone resistant *S*. *aureus* strain (Fig. 5C,E and Supplementary Fig. S3A,B). We took advantage of the fact that quinolones are known to act on DNA gyrase by blocking subunit A. Therefore, resistant variants have alterations in subunit A. As expected, ciprofloxacin did not inhibit the enzymatic activity of the resistant strains. UVI5008 inhibited the resistant strains and wild-type (WT) strains equally well (Fig. 5). We propose that these data suggest that UVI5008 is either targeting a different pocket in subunit A or targeting subunit B.

**Figure 6:**
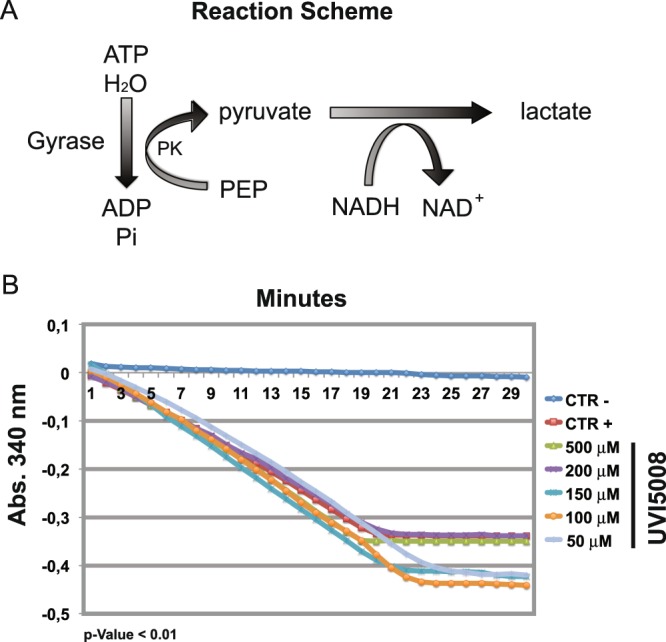
*S*. *aureus* gyrase ATPase linked assay. Schematic representation of enzymatic reaction **(A)**. Enzymatic evaluation of ATPase activity of *S*. *aureus* DNA gyrase in the presence of UVI5008 from 50 to 500 μM compared with the negative control (no enzyme) and the positive control (no inhibitor).

To show the direct activity of UVI5008 on subunit B, we assessed the ATPase activity of subunit B^32^ by DNA gyrase ATPase linked assay. This method correlates ATP hydrolysis with the conversion of NADH to NAD^+^. The NAD^+^ production is assessed at 340 nm and compared to the negative control (Fig. 6A). No alteration of the DNA gyrase ATPase activity (Fig. 6B) in the presence of UVI5008 was observed. These results indicate that UVI5008 is decreasing gyrase activity without interfering with the ATPase enzymatic activity. The precise mechanism of UVI5008 interference with gyrase activity was not determined by our experiments. Additional mechanistic studies will be necessary to unravel the molecular mechanism of action of UVI5008 on DNA gyrase activity.

### UVI5008 modifies MSSA and MRSA phenotypes

To determine whether UVI5008 acts only on DNA gyrase or if it has other microbial targets, we evaluated the effect of UVI5008 on *S*. *aureus* morphology via Scanning Electron Microscopy (SEM)^31^.

**Figure 7:**
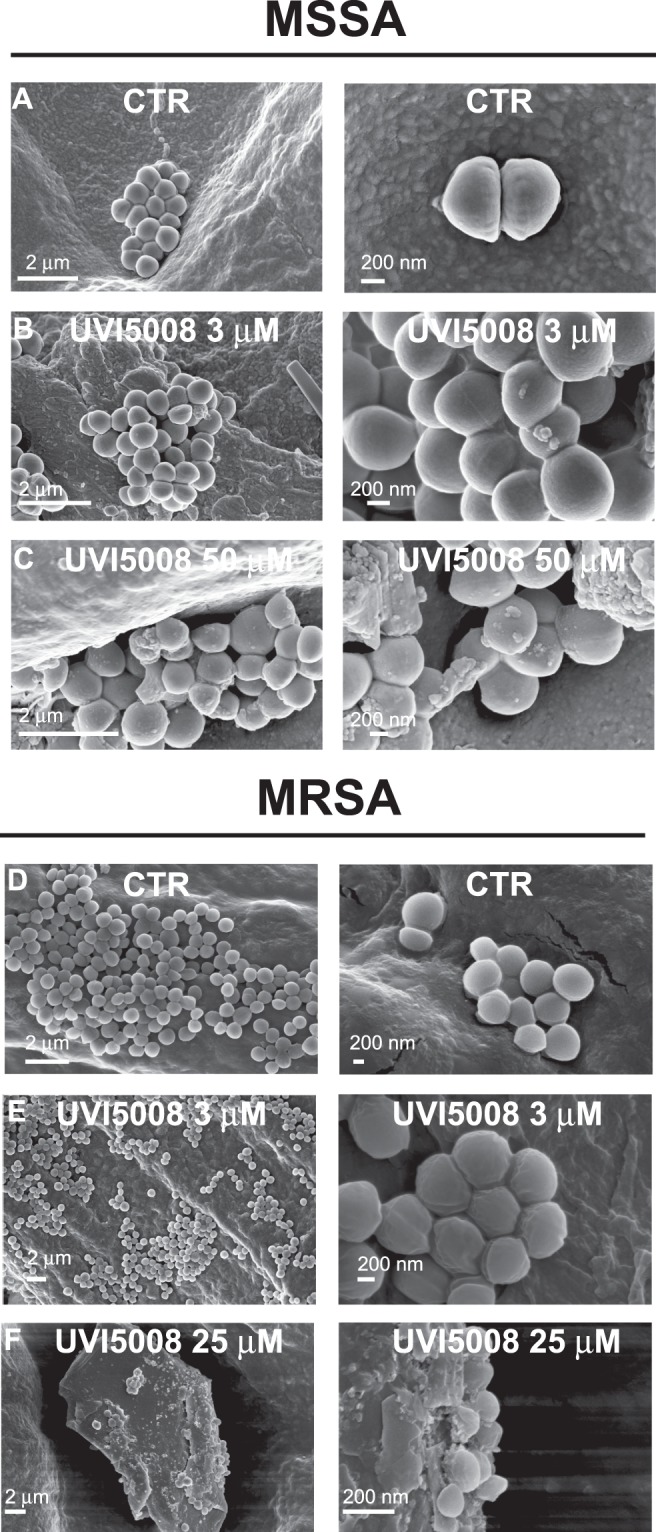
Two different magnifications of each surface morphology of MSSA ATCC and MRSA treated with UVI5008 at 3 μM and 50 μM compared to control, acquired via scanning electron microscopy.

In our experiments so far, we have highlighted DNA gyrase inhibition activity. Here, we also investigated the surface integrity in response to UVI5008 treatment. Bacteria treated with solvent (mock) (Fig. 7A), ampicillin (Supplementary Fig. S4A), Psa_A (Supplementary Fig. S4B,C) and UVI5008 were observed by SEM. No detectable alterations were observed for the ampicillin, Psa_A or mock treated bacteria. The UVI5008 treated bacteria showed drastic phenotypic modifications on their surface (Fig. 7A,F).

Following 20 hours of treatment with 50 μM UVI5008, the SEM data showed marked cell wall damages. These damages are more evident at higher concentrations, but signs of cell damage with irregular surface and dents are already present even at a concentration of 3 μM Control cells showed a smooth and flawless external surface. The total number of cells in the treated samples was also diminished compared to the control. This could be a combined effect of the drug on bacterial growth and on the size of single cells. A similar effect was observed for both MSSA and MRSA strains. These observations lead to the possibility that the effects mediated by UVI5008 treatment could involve modification, alteration and regulation.

## Discussion

Bacterial antibiotic resistance represents an open battlefield. Currently, the time between the launch of a new antibiotic on the market and the acquisition of resistance is dramatically shortened^33^. As a result, new antibacterial molecules are urgently needed. In the present study, we report for the first time the evaluation of a panel of epigenetic modulators against Gram-negative and Gram-positive bacteria. Our investigated compound, UVI5008, inhibits *S*. *aureus* growth and exhibits bacteriostatic properties. The lack of activity on Gram-negative bacteria could be attributed to their cell-wall complexity. We observed different UVI5008 inhibition curves for diverse *S*. *aureus* strains. The results showed that all biofilm producer strains were more resistant to UVI5008 treatment. Biofilms are aggregates of microbial cells associated with an inert or living surface and included in a self-produced extracellular polymer matrix^34^. Biofilm is mainly composed of polysaccharides, proteins, extracellular DNA and lipids^35^. According to *Cori et al*., bacteria within the biofilm are less susceptible to antimicrobial agents^36^. Reduced susceptibility is often due to the limited spread of drugs through the biofilm polymer matrix^37^ and to physiological changes (cell growth and the metabolic state) in bacteria due to environmental conditions that characterize the biofilm^38^. The lower potency in biofilm could indeed be attributed to both (i) the decreased UVI5008 diffusion within the biofilm and (ii) the lower metabolic rate of the bacteria in the biofilm.

UVI5008 showed discrete biofilm disaggregation activity at a concentration of 25 μM. The same concentration was necessary to achieve total growth inhibition for multi-resistant *S*. *aureus* strains.

The SAR study suggests that the full UVI5008 structure is necessary to preserve its antibacterial activity. Most of the analogues produced for the SAR study were unable to inhibit bacterial growth. The elongation of the hydrocarbon chain reduces the activity of the compound, and the symmetrical dimer with the disulphide bridge is of utmost importance. Only replacement of bromine by other large halogens could retain antibacterial activity, albeit with reduced efficacy. This is supported by our data, as the only two compounds consistently reaching inhibition of 60–80% of bacterial growth are UVI5042 and UVI5043, which display Cl and I in place of Br, respectively. Future work will attempt to design and test new analogues to further improve the antimicrobial activity.

To uncover mechanism of action and target(s) of UVI5008, we explored morphological damage and inhibition of gyrase activity. Previous results showed that Psa_A, a natural derivative of UVI5008, acts as an inhibitor of the DNA gyrase enzyme of *S*. *aureus*. DNA gyrase consists of two A subunits and two B subunits. The A subunits are responsible for negative supercoiling of the DNA, while the B subunits provide the energy required for activity by ATP hydrolysis. We have shown here that UVI5008 reduced more than 80% of the *S*. *aureus* gyrase activity. Despite the data obtained, UVI5008 did not inhibit the enzymatic activity of subunit B. These results, while inconclusive on the precise mechanism of action, led us to hypothesize that UVI5008 may interact with a different region of subunit B. Alternatively, UVI5008 may interact with subunit A, but our data on the subunit A resistant variants would imply that any potential activity on subunit A is on a different region compared to previously identified inhibitors. The molecular mechanism of gyrase activity inhibition remains unclear and will be further investigated in future work.

Here, we showed that UVI5008 has anti-gyrase activity, not only for WT strains but also for previously identified mutants. This is significant because when UVI5008 and the previous drugs are combined as a cocktail, developing resistance to the cocktail may be avoided. In future work, we will address this specific hypothesis and, if resistance is elicited, may be able to shed light on the precise mechanism of action of UVI5008. Moreover, UVI5008 treatment of MSSA generated a morphological modification on the bacterial surface. Disruption of the cell wall was observed by SEM analysis, where, after treatment, the presence of rough surfaces, cavities, blebs and alteration of normal shape were recorded. In addition, we could detect an overall depletion of viable cell count with an accumulation of cell debris. Aberrant structures were produced on bacterial cell walls after treatment with UVI5008. Similar surface structures have been recently described by *Walker J*.*N*. *et al*., who showed a large spike-like protein called the “Giant Staphylococcal Surface Protein” (GSSP) on the surface of *S*. *aureus*^39^. The presence of GSSP on the *S*. *aureus* cell surface altered the ability to induce agglutination with a significant reduction in terms of pathogenesis. Previous studies^26^ in xenograft models showed that treatment with UVI5008 once a day for 7 days daily at 40 mg/kg was not toxic for eukaryotic cells. Taken together, these findings suggest a good therapeutic window for UVI5008 treatment in *S*. *aureus* infection at a concentration of 7 mg/L (40 μM), which totally inhibits bacterial growth. These data suggest that UVI5008 has potential for translational application and could be a valid compound for alternative MRSA treatment.

## Materials and Methods

### Bacterial strains

The strains used for the antimicrobial assays were *E*. *coli* strain ATCC 11219, *Klebsiella pneumoniae* strain ATCC 10031, *Enterococcus faecalis* strain ATCC 29212, *Staphylococcus aureus* strain ATCC 6538, a multi-sensitive clinical strain of *S*. *aureus*, a Met.R clinical strain of *S*. *aureus*, a macrolide resistant clinical strain of *S*. *aureus* and a β-lactamase producer strain. To standardize the bacterial cell suspension for the antibacterial activity assay, fresh colonies of each strain cultured on Mueller-Hinton agar (MHA) plates were used to inoculate Mueller-Hinton broth (MHB) medium and grown at 37 °C in a shaking incubator overnight (O.N.). Following O.N. growth, the bacterial suspension was transferred to fresh medium and further incubated at 37 °C until log-phase growth was reached (1 × 10^8^ CFU/ml). Serial dilution and subsequent plating were performed to determine the final concentration of bacteria (1 × 10^6^ CFU/ml).

### Antimicrobial activity assays

Susceptibility testing was performed following the broth microdilution method outlined by the National Committee on Clinical Laboratory Standards (NCCLS) using sterile 96-well microliter plates. Compound concentrations were selected based on their known activity as epigenetic modulators. Two different bacterial species for each group, based on the Gram classification, were selected: for Gram-negative bacteria, we selected *Escherichia coli* (*E*. *coli* ATCC CRM-11229) and *Klebsiella pneumoniae* (*K*. *pneumoniae* ATCC 10031); for Gram-positive bacteria, we selected *Staphylococcus aureus* (*S*. *aureus* ATCC 6538) and *Enterococcus faecalis* (*E*. *faecalis* ATCC29212). The dilutions (1 to 100 μM) of each compound were prepared in brain heart infusion broth at a volume of 100 μl/well. Each well was inoculated with 50 μl of the standardized bacterial inoculum, corresponding to a final test concentration of approximately 5 × 10^5^ CFU/ml. The antimicrobial activities were expressed as the MIC, the lowest concentration of a compound at which 100% inhibition of microbial growth was observed after 4, 6 and 20 h of incubation at 37 °C. When a significant difference was observed, we performed a further set of triplicate antibacterial experiments using the compound at concentrations close to the average MIC obtained. All experiments were performed in triplicate, and the means ± standard deviations are reported.

### Biofilm production assay

*S*. *aureus* biofilm production was executed through the 2,3,5-triphenyl-tetrazolium chloride (TTC) reduction assay^40^. In short, biofilms were grown in sterile 96-well polystyrene flat bottom microtitre plates. Aliquots of 200 μl of bacterial suspension (1 × 10^5^ CFU/ml) in BHI medium were transferred to each well and incubated at 37 °C for 24 hours without shaking. After incubation, the supernatant fluids were gently removed, and the remaining non-adherent cells were washed twice with PBS. To visualize biofilm growth, 100 μl of the 1% TTC solution was added to each well and incubated for 3 hours at room temperature. The absorbance was measured at 600 nm using a microtiter plate reader.

### Biofilm degradation assay

The ability of UVI5008 to disrupt the *S*. *aureus* biofilm was evaluated by the reduction TTC assay. Briefly, 200 μl of bacterial suspension (1 × 10^5^ CFU/ml) was plated in each well of a 96-well plate and incubated at 37 °C for 24 hours to allow the formation of mature biofilm. After incubation, non-adherent cells were removed through two washes with PBS. UVI5008 and vancomycin at 200 μl, diluted in the used medium to concentrations of 50, 25, 10, 5 and 1 μM for UVI5008 and 10.4 μM for vancomycin, were added to the mature biofilm. To evaluate activity with a combination of drugs, preformed biofilm was incubated with UVI5008 alone and in combination with ampicillin, vancomycin and ciprofloxacin at concentrations of 50, 0.34, 2.6, and 3 μM. The positive and negative controls were represented by untreated biofilm producer (ATCC 25923) and non-producer (ATCC 6538) *S*. *aureus*, respectively. After 24 hours of incubation, planktonic cells were removed through two washes with PBS. To visualize biofilm growth, 100 μl of the 1% TTC solution was added to each well and incubated for 3 hours at room temperature. The absorbance was measured at 600 nm using a microtitre plate reader.

### Structure-activity relationships

The UVI5008 SAR compounds were described by Pereira R. *et al*. (2012). Compounds were dissolved at a concentration of 50 mM in DMSO and then tested via antimicrobial activity assays. The data were acquired and analysed via *Heatmapper*^41^. The cluster was generated based on the average linkage and the Euclidean distance measurement.

### *S*. *aureus* clinical isolates

All biological samples were collected following the rules of sterility in the “Vanvitelli” university hospital based in Naples at the University of Study of Campania Luigi Vanvitelli. The samples were analysed following the manufacturer’s instructions using the Phoenix instrument (Becton Dikinson). The antibiograms of isolated *S. aureus* strains were obtained following the EUCAST rules. Finally, the P88 panel was used for the Gram-positive bacteria.

### Enzymatic assays

Supercoiling DNA gyrase assays were performed using a *S. aureus* gyrase supercoiling assay kit according to the manufacturer’s instructions (Insiparlis cod. SAG4001). Reaction mixtures (30 μl) containing 40 mM HEPES-KOH (pH 7.6), 10 mM magnesium acetate, 10 mM DTT, 2 mM ATP, 500 mM potassium glutamate, 0.05 mg/ml albumin, 0.5 μg of relaxed pBR322 DNA, 1 U of DNA gyrase, and either 50, 25, 10, or 5 μM UVI5008 dissolved in 10% DMSO or 30 μM ciprofloxacin dissolved in 0.01 N HCl were incubated at 37 °C for 30 minutes. The negative and positive controls were represented by the absence and presence of the enzyme in 10% DMSO. The reaction was stopped with 30 μl of STEB (40% (w/v) sucrose, 100 mM Tris-HCl pH 8, 10 mM EDTA, 0.5 mg/ml bromophenol blue) and 30 μl of chloroform/isoamyl alcohol (v:v 24:1). DNA supercoiling was analysed by 1% agarose electrophoresis in the absence of ethidium bromide. The software ImageJ was used to quantify the signal present in the agarose gel, and the values were converted to the relative % of enzymatic activity. The IC_50_ was calculated by GraphPad Prism software.

The DNA gyrase ATPase linked assay was performed using a *S*. *aureus* gyrase ATPase linked assay kit according to the manufacturer’s instructions (Insiparlis cod.ATPSAG001). Reaction mixtures (100 μl) contained 40 mM HEPES-KOH (pH 7.6), 10 mM magnesium acetate, 10 mM DTT, 2 mM ATP, 500 mM potassium glutamate, 0.05 mg/ml albumin, 3 μg of relaxed pBR322 DNA, 80 mM PEP, stock pyruvate kinase/lactate dehydrogenase (reported concentration in the manufacturer’s instructions), 20 mM NADH, 50 nM DNA gyrase, and either 50, 25, 10, or 5 μM UVI5008 dissolved in 10% DMSO or 30 μM ciprofloxacin dissolved in 0.01 N HCl. The negative and positive controls were represented by the absence and presence of the enzyme in 10% DMSO, respectively. Absorbance at 340 nm was measured for 10 min at 25 °C. The reaction began after the addition of 30 mM ATP. Absorbance at 340 nm was then monitored for 60 minutes at 25 °C.

### Scanning electron microscopy

Morphological studies of the bacterial surface were performed by scanning electron microscopy. Cell suspensions were fixed in 4% paraformaldehyde in phosphate buffer, dehydrated in 30%, 50%, 75%, 90% and 100% ethanol three times for 5 min each (spin down in between) and dried in a critical point dryer (EMITECH K850) using a Swinnex filter holder. Filters were then mounted on stubs and sputter-coated with gold (Denton Vacuum Desk V) before observation under a scanning electron microscope (Supra 40 ZEISS; EHT = 5.00 kV, WD = 22 mm, detector in lens).

## Electronic supplementary material

Supplementary Figures 1-4
