# Supplementary material for: Epigenetic modulator UVI5008 inhibits MRSA by interfering with bacterial gyrase
Source: Sci Rep. 2018 Sep 3;8:13117. doi: 10.1038/s41598-018-31135-9 (PMC6120918; doi:10.1038/s41598-018-31135-9)

# Epigenetic modulator UVI5008 inhibits MRSA by interfering with bacterial gyrase

Gianluigi Franci, Veronica Folliero, Marcella Cammarota, Carla Zannella, Federica Sarno, Chiara Schiraldi, Angel R. de Lera, Lucia Altucci, Massimiliano Galdiero

**Supplementary material:**

Supplemental Table 1: List of compounds and their concentration used in the screening.

S.1 MBC assay evaluation on brain-heart plate disseminated with respective 50 µl of ATCC sample and MRSA in figure 2 A-B post 20h of growth.

S.2 Biofilm formation evaluation on ATCC SA compared with FI MSSA and resistant SAs (A). Degradation activity mediated by UVI5008 on FI MSSA (B).

S.3 Field isolate documents. Antibiogram and resistance identification for the two different field isolated *S. aureus* strains (A,B).

S.4 Surface morphology of MSSA ATTC and MRSA acquired via scanning electron microscope treated with Psa-A at 3 µM and 50 µM compared to positive (Amp) and negative (vehicle) controls.

| Name | Class | Concentrations |
| --- | --- | --- |
| **SAHA** | HDAC inhibitor | 0,5 µM–5,0 µM |
| **MS-275** | Class I HDAC inhibitor | 0,5 µM–5,0 µM |
| **UVI5008** | HDAC-HMT inhibitor | 0,5 µM–5,0 µM |
| **ATRA** | all-trans retinoic acid | 0,5 µM–5,0 µM |
| **VPA** | HAT inhibitor | 5,0 µM–100 µM |
| **EX-527** | SIRT1 inhibitor | 0,1 µM–1000 µM |
| **AGK-2** | SIRT2 inhibitor | 10 µM–1000 µM |
| **2,4-Pyridinedicarboxylic** | DNMT inhibitor | 10 µM–50 µM |
| **Ellagic Acid** | DNMT inhibitor | 10 µM–50 µM |
| **GSK-126** | EZH2 inhibitor | 0,5 µM–50 µM |
| **GSK-J4** | KDM inhibitor | 10 µM–50 µM |
| **PBLJA017** | Pro-Apoptotic drug | 5 µM–10 µM |

Supplementary Table 1.
Selected compound with their activity and the double concentration used.

Supplementary Figure 1.
MBC assay evaluation on brain-heart plate disseminated with respective 50 µl of ATCC sample and MRSA in figure 2 A-B post 20h of growth.

Epigenetic modulator UVI5008 mechanism of action against MRSA
Gianluigi Franci, Veronica Folliero, Marcella Cammarota, Carla Zannella, Federica Sarno, Chiara Schiraldi, Angel R. de Lera, Lucia Altucci, Massimiliano Galdiero.

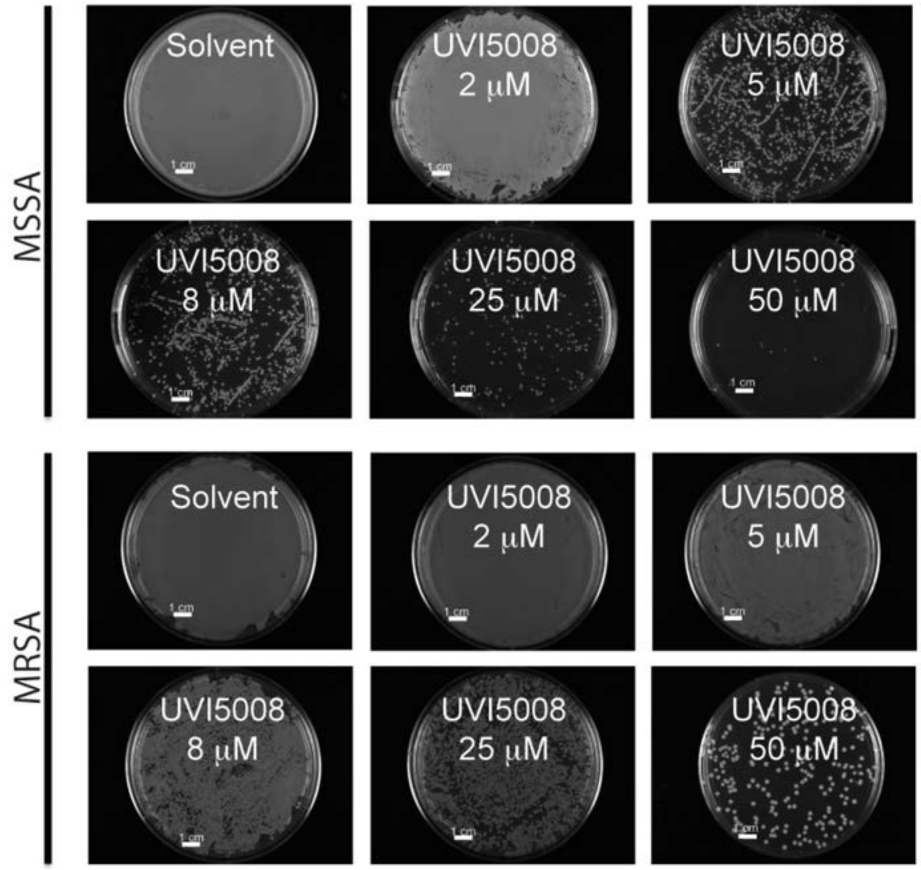

Supplementary Figure 2.
Biofilm formation evaluation on ATCC SA compared with FI MSSA and resistant SA (A).
Degradation activity mediated by UVI5008 on FI MSSA (B).

Epigenetic modulator UVI5008 mechanism of action against MRSA
Gianluigi Franci, Veronica Folliero, Marcella Cammarota, Carla Zannella, Federica Sarno, Chiara Schiraldi, Angel R. de Lera, Lucia Altucci, Massimiliano Galdiero.

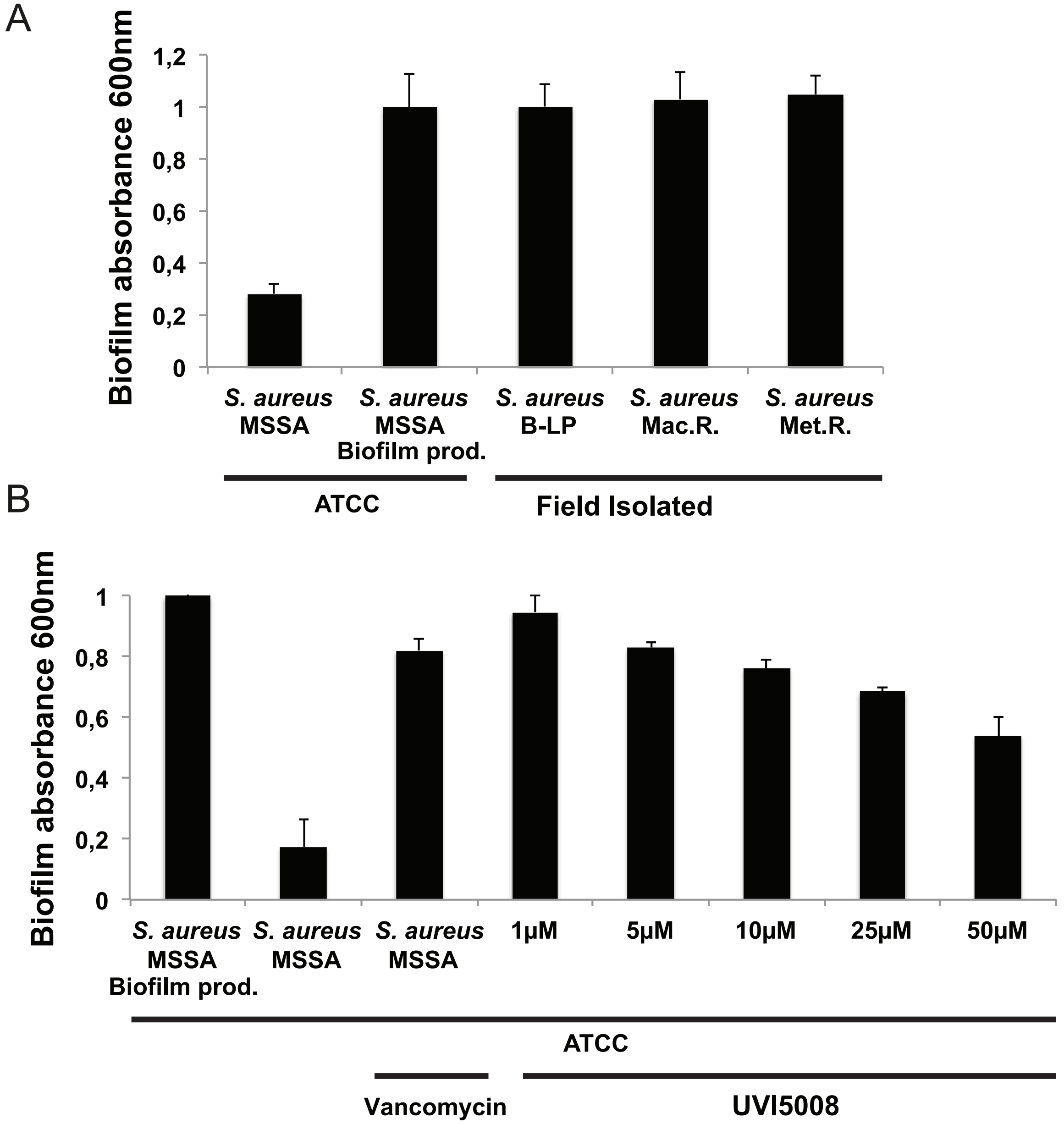

Supplementary Figure 3. Field isolate documents. Antibiogram and resistance identification for the two different Quinolone Resistance (Quin. R.1 and 2) field isolated S. aureus strains (A,B).

A Quin. R. 1

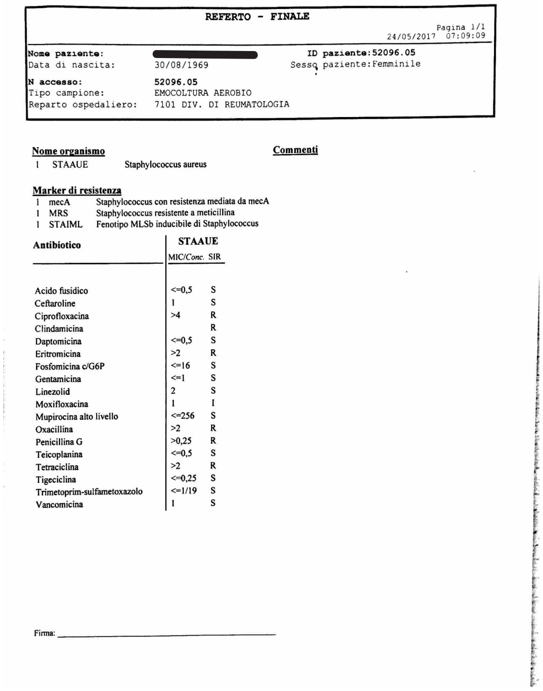

**REFERTO - FINALE**

Pagina 1/1
24/05/2017 07:09:09

**Nome paziente:** [redacted] **ID paziente:** 52096.05
Data di nascita: 30/08/1969 Sesso paziente: Femminile
**N accesso:** 52096.05
Tipo campione: EMOCOLTURA AEROBIO
Reparto ospedaliero: 7101 DIV. DI REUMATOLOGIA

**Nome organismo** **Commenti**
1 STAAUE Staphylococcus aureus

**Marker di resistenza**
1 mecA Staphylococcus con resistenza mediata da mecA
1 MRS Staphylococcus resistente a meticillina
1 STAIML Fenotipo MLSb inducibile di Staphylococcus

| Antibiotico | STAAUE MIC/Conc. | SIR |
|---|---|---|
| Acido fusidico | <=0,5 | S |
| Ceftaroline | 1 | S |
| Ciprofloxacina | >4 | R |
| Clindamicina | | R |
| Daptomicina | <=0,5 | S |
| Eritromicina | >2 | R |
| Fosfomicina c/G6P | <=16 | S |
| Gentamicina | <=1 | S |
| Linezolid | 2 | S |
| Moxifloxacina | 1 | I |
| Mupirocina alto livello | <=256 | S |
| Oxacillina | >2 | R |
| Penicillina G | >0,25 | R |
| Teicoplanina | <=0,5 | S |
| Tetraciclina | >2 | R |
| Tigeciclina | <=0,25 | S |
| Trimetoprim-sulfametoxazolo | <=1/19 | S |
| Vancomicina | 1 | S |

Firma: ______________________

B Quin. R. 2

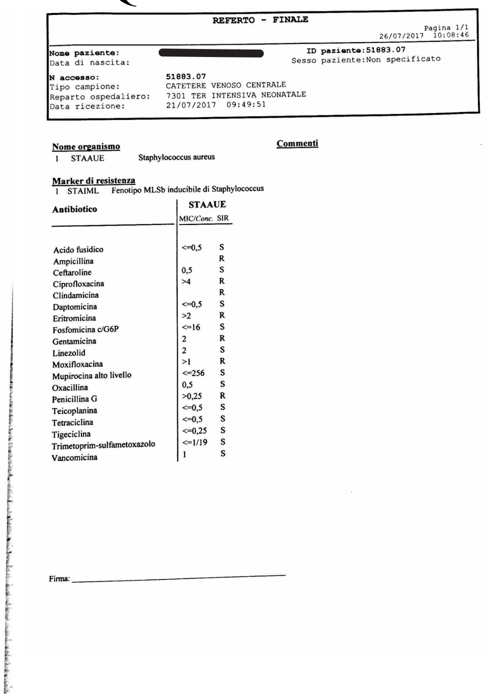

**REFERTO - FINALE**

Pagina 1/1
26/07/2017 10:08:46

**Nome paziente:** [redacted] **ID paziente:** 51883.07
Data di nascita: Sesso paziente: Non specificato
**N accesso:** 51883.07
Tipo campione: CATETERE VENOSO CENTRALE
Reparto ospedaliero: 7301 TER INTENSIVA NEONATALE
Data ricezione: 21/07/2017 09:49:51

**Nome organismo** **Commenti**
1 STAAUE Staphylococcus aureus

**Marker di resistenza**
1 STAIML Fenotipo MLSb inducibile di Staphylococcus

| Antibiotico | STAAUE MIC/Conc. | SIR |
|---|---|---|
| Acido fusidico | <=0,5 | S |
| Ampicillina | | R |
| Ceftaroline | 0,5 | S |
| Ciprofloxacina | >4 | R |
| Clindamicina | | R |
| Daptomicina | <=0,5 | S |
| Eritromicina | >2 | R |
| Fosfomicina c/G6P | <=16 | S |
| Gentamicina | 2 | R |
| Linezolid | 2 | S |
| Moxifloxacina | >1 | R |
| Mupirocina alto livello | <=256 | S |
| Oxacillina | 0,5 | S |
| Penicillina G | >0,25 | R |
| Teicoplanina | <=0,5 | S |
| Tetraciclina | <=0,5 | S |
| Tigeciclina | <=0,25 | S |
| Trimetoprim-sulfametoxazolo | <=1/19 | S |
| Vancomicina | 1 | S |

Firma: ______________________

Supplementary Figure 4.
Surface morphology of MSSA ATTC and MRSA acquired via scanning electron microscope treated with Psa-A at 3 μM and 50 μM compared to positive (Amp) and negative (vehicle) controls.

Epigenetic modulator UVI5008 mechanism of action against MRSA

Gianluigi Franci, Veronica Folliero, Marcella Cammarota, Carla Zannella, Federica Sarno, Chiara Schiraldi, Angel R. de Lera, Lucia Altucci, Massimiliano Galdiero.

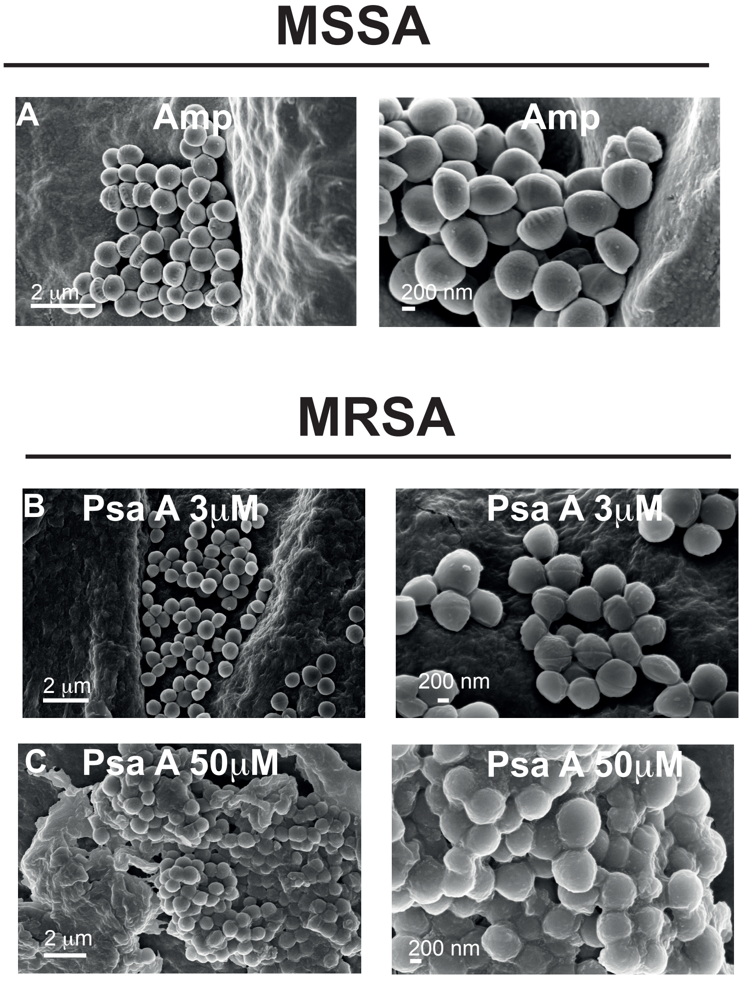

Supplement: Supplementary file 1 — Supplementary Figures 1-4 [file 41598_2018_31135_MOESM1_ESM.pdf]
